# Preparing patients according to their individual coping style improves patient experience of magnetic resonance imaging

**DOI:** 10.1007/s10865-022-00361-y

**Published:** 2022-09-08

**Authors:** Janika E. M. Madl, Sarah C. Sturmbauer, Rolf Janka, Susanne Bay, Nicolas Rohleder

**Affiliations:** 1 Chair of Health Psychology, Friedrich-Alexander-Universität Erlangen-Nürnberg, Nägelsbachstr. 49a, 91052 Erlangen, Germany; 2 Department of Radiology, University Hospital Erlangen, Maximiliansplatz 1, 91054 Erlangen, Germany; 3 Siemens Healthcare GmbH, Allee am Röthelheimpark 21, 91052 Erlangen, Germany

**Keywords:** Patient experience, Anxiety, Cortisol, Magnetic resonance imaging, Patient preparation, Coping style

## Abstract

MRI-related anxiety is present in 30% of patients and may evoke motion artifacts/failed scans, which impair clinical efficiency. It is unclear how patient anxiety can be countered most effectively. Habitual preferences for coping with stressful situations by focusing or distracting one’s attention thereof (coping style) may play a key role in this context. This study aimed to compare the effectiveness of two patient-preparation videos with informational vs. relaxational content and to determine whether the fit between content and coping style affects effectivity. The sample consisted of 142 patients (*M* = 48.31 ± 15.81 years). Key outcomes were anxiety, and cortisol as a physiological stress marker. When not considering coping style, neither intervention improved the patients’ reaction; only patient preparation that matched the patients’ coping style was associated with an earlier reduction of anxiety. This suggests that considering individual preferences for patient preparation may be more effective than a one-size-fits-all approach.

## Introduction

Magnetic resonance imaging (MRI) is a widely used diagnostic imaging method and generally well-tolerated (Dantendorfer et al., 1997; Mackenzie et al., 1995; Madl et al., 2022). Still, multiple studies have shown that MRI does induce stress and anxiety in up to one third of all patients; this can be seen in both psychological and physiological markers (Carlsson & Carlsson, 2013; Eatough et al., 2009; Eshed et al., 2007; Forshaw et al., 2018; Madl et al., 2022; Peters et al., 2011). Anxiety and stress in patients, in turn, may have a negative impact on clinical processes: A negative patient experience has been linked to a higher prevalence of motion artifacts and thus scan repetitions, longer scan duration, and more premature terminations (Dantendorfer et al., 1997; Dewey et al., 2007; Enders et al., 2011; Madl et al., 2022; Nguyen et al., 2020; Powell et al., 2015). Andre et al. (2015) calculated $115,000 lost revenue per scanner every year due to unexpected patient behavior, which at least partially seems to trace back to anxiety in patients.

Different approaches have been taken to reduce MRI-related anxiety and improve patient experience. Informational interventions aim to reduce feelings of uncertainty which can burden MRI patients (Carlsson & Carlsson, 2013; Mackenzie et al., 1995). However, such purely informational interventions have revealed mixed results (Munn & Jordan, 2013): While some authors found no or even a detrimental effect of additional information on anxiety (Quirk et al., 1989; Törnqvist et al., 2006), others did find the expected reduction of anxiety (Selim, 2001; Youssefzadeh et al., 1997). In contrast to that, other interventions aim to enhance the patients’ ability to cope with the aversive situation, for instance through relaxation, distraction, or reappraisal of the situation (Krohne & de Bruin, 1998; Miller et al., 1989), and have been shown to effectively reduce anxiety in patients; yet, the reported effect sizes vary considerably (Munn & Jordan, 2013).

Interindividual differences in personality traits may contribute to explaining these inconsistencies. In the broader field of medical procedures, the Model of Coping Modes (Krohne, 1993, 2003) has provided insights into this matter. It proposes that people differ in their habitual way of coping with stress-inducing situations; the two dimensions vigilance and cognitive avoidance describe how attention shifts when being confronted with aversive threats. Vigilance is characterized by an increased focus on aversive cues in order to reduce the uncertainty or ambiguity of a threatening situation (e.g., by searching for information or anticipating possible negative outcomes). Cognitive avoidance, on the contrary, means a tendency to distract attention from threatening cues in order to reduce the emotional arousal induced by these cues; this may be achieved via distraction or trivialization of the situation (Krohne, 1993, 2003). Depending on the individual manifestation of these dimensions, four coping styles can be differentiated (De Bruin et al., 2001; Krohne, 1993, 2003): Individuals of the coping style repression (low vigilance, high cognitive avoidance) generally aim to reduce the emotional arousal induced by threatening cues through diverting their attention from those. Rather than trying to generate a valid expectation of the threatening situation, they tend to disavow its’ aversive characteristics. Sensitization (high vigilance, low cognitive avoidance) is characterized by increased attention towards threatful cues in order to create a realistic picture of what to be expected and avoid “negative surprises”. By doing so, the uncertainty that renders a situation stressful can be reduced. Individuals with high scores on both dimensions (high vigilance, high cognitive avoidance) are considered highly anxious individuals. This coping pattern is characterized by a low tolerance for arousal as well as uncertainty; this evokes fluctuating coping behavior, as neither employment of vigilant nor avoiding coping strategies can meet all needs and both strategies cannot be employed simultaneously. Last, individuals with low scores on both dimensions (low vigilance, low cognitive avoidance) are postulated to tolerate uncertainty as well as arousal relatively well. This results in a non-defensive coping style which enables them to vary their coping strategy adaptively depending on the situational requirements (De Bruin et al., 2001; Krohne, 1993, 2003).

Findings on the sources of MRI-related anxiety support the hypothesis of different coping styles and patient needs. To begin with, uncertainty and lack of information seems to constitute a decisive factor for some patients, who report fear of being harmed by the machine and fear of pain (Carlsson & Carlsson, 2013; Dantendorfer et al., 1997; Katz et al., 1994; Thorpe et al., 2008). These fears seem to trace back to lacking knowledge and low tolerance of the related feelings of uncertainty, which may be founded in a vigilant coping style. Providing appropriate information could be suitable to address these concerns. Apart from these aspects, some patients have also reported to fear the physical properties of MRI (e.g. noise, duration, constriction), and to worry about the results to be expected from the examination and their health (Carlsson & Carlsson, 2013; Dantendorfer et al., 1997; Katz et al., 1994; Thorpe et al., 2008). These fears could trace back to a low tolerance for the arousal induced by MRI-related threatening cues, i.e., an avoidant coping style. The physical properties and potentially threatening results are inherent and unalterable characteristics of MRI. Accordingly, additional information does not seem to be capable of reducing these fears appropriately. Instead, a successful coping strategy could lie in distracting one’s attention thereof.

In the context of invasive diagnostic procedures and surgeries, preparing patients with interventions that are congruent with their coping style has been shown to produce desirable effects, especially for repressers and sensitizers. These groups are characterized by a clear preference for one coping strategy over the other which is why the clearest hypotheses can be formulated. Addressing the preferred strategy should meet their needs, whereas the situation is less clear when patients have a high or low tendency for both strategies like highly anxious and non-defensive individuals. Patients who strongly strive for threat-relevant information have been shown to search for more online health information (de Looper et al., 2021), to ask more questions in medical consultations—especially in highly-threatening situations (Ong et al., 1999; Timmermans et al., 2007), and to have a higher interest in learning about own cancer risk (Dahle Ommundsen et al., 2022). The opposite pattern is found for patients who strive to avoid threatening information: They tend to ask less questions which results in shorter consultations (Ong et al., 1999; Timmermans et al., 2007). Accordingly, patient preparation congruent with coping style has been associated with lower levels of behavioral indices of pain and self-reported anxiety, as well as shorter recovery times and better adaptation in patients undergoing coloscopy (Morgan et al., 1998), cardiac catheterization (Ludwick-Rosenthal & Neufeld, 1993), and oral surgery (Martelli et al., 1987). Krohne and El-Giamal (2008) found a significant increase of anxiety on the day of surgery when highly avoidant / vigilant patients had been prepared with information / relaxation; this increase was not visible when the intervention met the patients’ avoidant or vigilant needs. Similarly, de Rooij et al. (2019) found beneficial effects of detailed information provided in the context of a survivorship care plans only for those who sought for information about their disease, but to have detrimental effects for those who didn’t.

In sum, the different root causes for MRI-related anxiety suggest that patients have different needs to cope with the situation successfully. Building upon the findings regarding the interaction of patient preparation and coping style in other medical contexts, it is thus conceivable that the patients’ coping style may also impact the effectivity of interventions to prepare patients for MRI.

We aimed to answer two questions with this study:

1. Do informational and relaxation interventions improve the patients’ psychological and physiological reaction to MRI? If so, we expect lower levels of anxiety and physiological stress in the information and relaxation group after the interventions than before and compared to the control group.
2. Will the effect be most pronounced for patients who are prepared congruently with their habitual coping style; that is repressers receiving a relaxational intervention to distract them from the upcoming MRI examination and sensitizers receiving an informational intervention to decrease uncertainty? If so, we expect a stronger reduction of anxiety and physiological stress after the interventions in the congruent groups than in the incongruent and the control group.

## Material and methods

### Study design and procedure

**Fig. 1:**
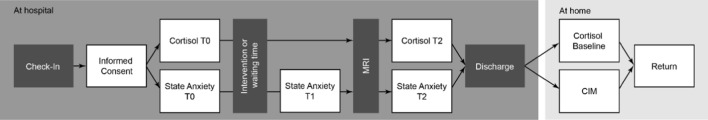
Study design. *Notes* Patients of the information and relaxation group received the respective intervention; patients of the control group waited for a comparable amount of time. CIM: Coping Inventory for Medical Situations (Sturmbauer et al., 2019)

This study was conducted at the Radiology Department of the University Hospital Erlangen from March to July 2021. The study employed a randomized controlled design; randomization was conducted prior to approaching patients via Robust Randomization App (RRApp, Version 3.0.1; Clinical Research Apps, 2017). Figure 1 provides an overview about the study design. The three study arms (information, relaxation, control) were distinguished and stratification for sex was applied. Patients were approached after check-in and while awaiting their MRI examination in the waiting room. After written informed consent, the current psychological state and basic demographics were assessed in a first questionnaire (T0) and patients provided the first saliva sample. Then, patients in the two interventional groups watched an informational or relaxational video (approx. 9 min) on a tablet with headphones; patients of the control group kept on waiting in the waiting room for the same time. Afterwards, the psychological state was re-assessed (T1). Upon completion of the MRI examination, patients provided the second saliva sample, and completed the third questionnaire (T2) which again measured the current psychological state. Upon leaving, a fourth questionnaire and two additional saliva sampling devices were handed out to the patients together with a stamped addressed envelope. Patients were instructed to answer the questionnaire within one week and provide two further saliva samples as reference values. These were supposed be obtained on a usual day at the same times as the pre- and post-MRI sample.

### Participants

Patients were eligible if they were outpatients receiving a head-first MRI examination. We only included patients scheduled after 1 pm to omit the most pronounced cortisol-changes in the morning; there was no cut-off time regarding end of data acquisition. Exclusion criteria were minority of age, insufficient language skills, pregnancy or nursing, and lack of time during the clinical workflow.

The sample size for this study was determined based on a power analysis regarding the main effect of the interventions on the patients’ anxiety. The analysis revealed that an ANOVA with 3 groups with an estimated *η*^2^ = 0.08, *α* = 0.05, and statistical power of 0.80, would require a sample size of *n* = 38 per group, adding up to a total sample size of *N* = 114. We further estimated a drop-out rate of approx. 25% and therefore adjusted the aim to *N* = 150, that is *n* = 50 per group. Of 150 patients recruited, three withdrew consent, two were excluded due to changes in the clinical workflow that prevented completion of the pre-MRI measures, and three due to receiving feet-first scans. This resulted in a final sample of 142 patients (age: *M* = 48.31 ± 15.81 years, range: 18–81 years; sex: 49.3% female, 50.0% male, 0.7% diverse). Of these, 50 received the informational intervention, 44 the relaxation intervention, and 48 no intervention (control group). Of all patients, 112 completed the home-questionnaire and took the saliva samples. Patients were referred to MRI for a broad range of clinical reasons, ranging from routine check-ups, over confirmation of tentative diagnoses (e.g. cancer, inflammatory diseases, degenerations), staging of disease, and aftercare. No patient received functional MRI (fMRI). Characteristics of the sample can be found in Table 1.

**Table 1:** Patient characteristics

**MRI scanner**	Aera: 74 (52.1%)	Vida: 63 (44.4%)	Sola: 5 (3.5%)
**Position**	Prone: 26 (18.3%)	Supine: 116 (81.7%)	
**Previous MRI-experience**	Yes: 117 (82.4%)	No: 16 (12.0%)	
**Intake of anxiolytika**	Yes: 5 (3.5%)	No: 137 (96.5%)	
**Examined body part**	Head and neck: 25 (17.6%) Breast: 13 (9.2%) Extremities: 21 (14.8%) Whole body: 2 (1.4%)	Spine: 23 (16.2%) Inner organs: 48 (33.8%) Heart: 10 (7.0%)	

*N* = 142

### Experimental conditions

#### Information

Patients of the information group watched an informational video of about 9 min length after finishing the first measures. The video was designed on the basis of previous findings and recommendations to provide information on the temporal sequence of the examination, the procedures, and information on the sensorics to be expected (Carlsson & Carlsson, 2013; Grey et al., 2000; Hjelm-Karlsson, 1991; Wallace, 1984). Therefore, the video demonstrated the entire procedure of receiving an MRI examination. It started with check-in at the healthcare provider and waiting and then showed the preparation for the MRI examination (e.g., taking off all metal objects) as well as the MRI examination itself. Thereby, the functioning of MRI was explained in a simple manner, also covering the noises and visuals to be expected during the examination. After showing the patient leaving the examination room and getting dressed again, the video ended with the patient leaving the hospital.

#### Relaxation

For the relaxation video we chose to use a self-hypnotic intervention; it’s length of 9 min was comparable to the information video. Hypnosis has been proven to be an effective instrument in a broad variety of medical contexts such as reducing pain, anxiety, and procedural times (Lang et al., 2000, 2006, 2008; Revenstorf & Peter, 2015; Tefikow et al., 2013). Thereby, hypnosis through a live hypnotist seems to be as effective as taped interventions (Tefikow et al., 2013). Our self-hypnotic video was specifically targeted at MRI patients. It consisted of a short induction and deepening through suggestions and breathing. Patients were suggested to experience sensations of calmness and relaxation, which should be transferred to the examination through posthypnotic suggestions before the reorientation phase. The audio file was accompanied by relaxing nature scenes; this was done to cater those patients who felt uncomfortable closing their eyes and to ensure comparability with the information intervention which also contained auditory as well as visual stimuli.

Both videos were evaluated in a small pilot study before the study-start and slightly adjusted according to the patients’ feedback. More detail on both interventions is provided via the TIDieR framework (Hoffmann et al., 2014; see Online Supplement).

#### Control

Patients of the control group did not receive an intervention. Instead, they kept on waiting in the waiting room after providing the first measures for a comparable amount of time as the interventional groups (approx. 9 min).

### Measures

#### Psychological outcomes

The psychological state was assessed via a German 5-item state only version of the *State-Trait-Anxiety-Inventory* (STAI-SKD; Englert et al., 2011) which economically assesses the transient emotional state of state anxiety with five items on a 4-point scale (e.g., “I am nervous”; 1 = *not at all*, 4 = *very much*). A mean score was calculated; higher values reflect higher levels of state anxiety. Additionally, we applied two Visual Analogue Scales (VAS) on the two facets of anxiety (Krohne, 2010; Liebert & Morris, 1967): agitation and worry (0 = *not agitated*/*worried at all*; 10 = *very agitated*/*worried*). The psychological state was assessed at T0, T1, and T2.

#### Physiological outcome

Salivary cortisol was acquired as a physiological stress indicator (Kirschbaum & Hellhammer, 1994). Saliva samples were obtained pre- and post-MRI (T0, T2) as well as at the same times on a comparison day (see Fig. 1) by means of “salivette” saliva sampling devices allowing collection of saliva with a polystyrol absporptive swab (Sarstedt, Nümbrecht, Germany). Salivettes were stored at − 20 °C or colder after collection. Participants were instructed to move the swab back and forth in their mouth for two minutes without chewing on it. The analysis was conducted as previously described for example in Becker and Rohleder (2020). Immediately prior to analysis, salivettes were thawed at room temperature and then centrifuged at 2000 g and 20 °C for ten minutes. Chemiluminescence immunoassays (CLIA, IBL, Hamburg, Germany) were used to determine salivary cortisol concentrations in duplicate. Intra- and inter-assay coefficients of variation were below 10%.

#### Coping style

Among other personality traits, the questionnaire that was filled in at home (see Fig. 1) assessed the patients’ coping style using the Coping Inventory for Medical Situations (CIM, [German: Angstbewältigungsinventar für medizinische Situationen, ABI-MS]; Sturmbauer et al., 2019). This situation-response-inventory assesses the patient’s habitual preferences to employ vigilant or avoidant coping strategies in the medical context. Four threatening medical situations with eight response options each (four vigilant, four avoidant) are described; patients are asked to indicate whether they would engage in any of these behavioral options on a dichotomous scale (0 = *no*, 1 = *yes*). A sum score for vigilance and cognitive avoidance across the four situations is calculated; higher values reflect a higher tendency to engage in the respective strategy.

#### Supplementary variables

Apart from these outcomes, we also assessed demographics and other variables via the questionnaires applied at T0 and T2. These, however, are not part of the current manuscript and therefore not displayed in detail. Data on the clinical condition and examination was recorded by the researchers as indicated by the respective technician in charge, who was blind for group assignment.

#### MRI scanners

All patients were examined on a 1.5 T or 3 T scanner (Magnetom Aera: 1.5 T, 70 cm diameter, 145 cm length; Magnetom Vida: 3 T, 70 cm diameter, 186 cm length; Magnetom Sola: 1.5 T, 70 cm diameter, 157 cm length; Siemens Healthcare, Erlangen, Germany).

### Statistical analyses

We first applied an rmANOVA with two within-subject factors (time: T0 vs. T2; day: day of MRI vs. at home) to analyze the individual cortisol response. Differences of the psychological and physiological stress measures between the different experimental groups were analyzed using separate mixed ANOVAs for each outcome; time (sampling point) was included as within-subjects factor and experimental group as between-subjects factor. When the *p*-values for the interaction effects were below 0.10, we conducted a separate rmANOVA for each group, using time (sampling point) as within-subjects factor.

**Fig. 2:**
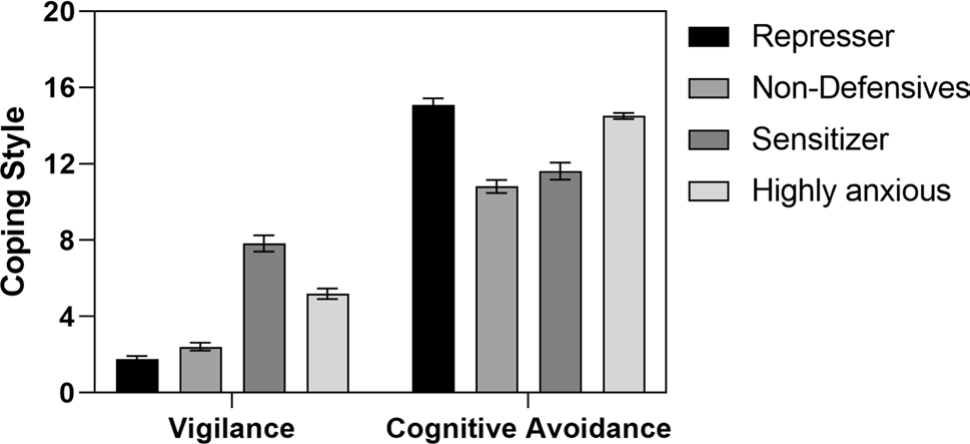
Coping strategies of the four clusters

Coping styles were determined on basis of the two CIM scales vigilance and cognitive avoidance (Sturmbauer et al., 2019) using hierarchical cluster analysis (Ward-method, euclidean distance; Fig. 2). Based on their mean values for the strategies, cluster 1 was defined as Repressers, cluster 2 as Non-Defensives, cluster 3 as Sensitizers and cluster 4 as Highly Anxious. Table 2 depicts the distribution of the coping styles to the three experimental groups.

**Table 2:** Distribution of the different coping styles to experimental groups

Experimental group	Coping style				
	Represser	Non-defensive	Sensitizer	Highly anxious	Total
Information	6	17	**10**	6	39
Relaxation	**10**	8	5	8	31
Control	7	19	8	7	41
Total	23	44	23	21	111

Patients in congruent combinations of coping style*experimental group are displayed fat

Mixed ANOVAs were applied with time (sampling point) as within-subjects-factor and group and coping style as between-subject factors to analyze the development of the patients’ response in the different groups depending on their respective coping style. To increase statistical power, we combined patients who had been prepared congruently (repressers in relaxation group; sensitizers in information group) and incongruently (all other combinations) in a second step. We then applied mixed ANOVAs to examine the differences in the psychological and physiological stress measures between congruent vs. incongruent patient preparation vs. control group; time was included as within-subjects factor and congruence as between-subjects factor. Again, we conducted a separate rmANOVA for each group, using time (sampling point) as within-subjects factor, when *p*-values for the interaction effects were below 0.10.

All tests were conducted at a two-sided 5% significance level without adjustment for the number of comparisons with SPSS 26. Greenhouse Geisser corrected *p*-values are reported when assumption of sphericity was violated. Due to lack of normality and skewness of data, log-transformed values of cortisol levels were used for analyses.

One patient (information group) reported to not have watched the video. Therefore, the respective patient was included in the control group for analysis.

## Results

### Descriptive and baseline information

**Fig. 3:**
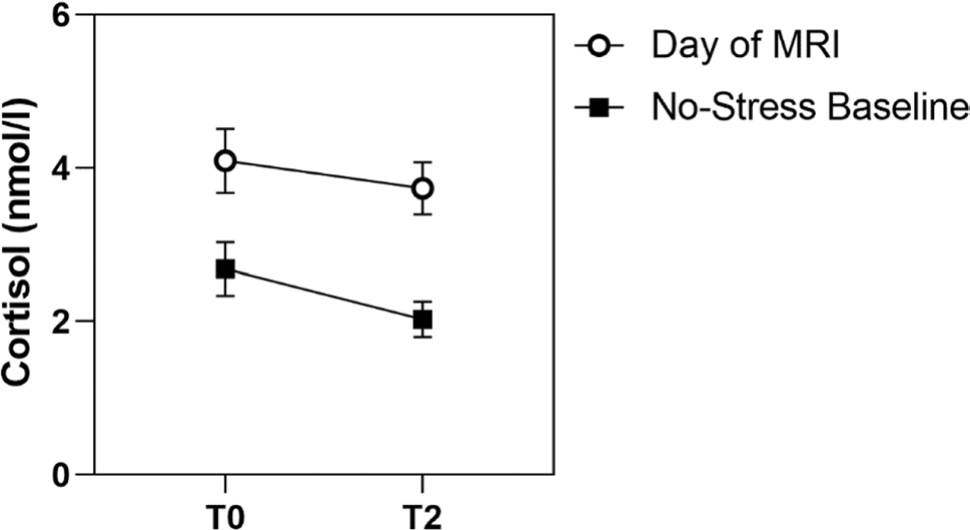
Cortisol levels on Day of MRI vs. No-Stress Baseline

Comparing cortisol levels measured at the day of MRI with the individual non-stress baseline, we found a significant main effect of time (F = 5.94, *p* = 0.016); cortisol levels decreased from T0 to T2, both at the day of MRI as well as on the comparison day. We also found a significant main effect of day (F = 41.94, *p* < 0.001): Cortisol levels were higher at the day of MRI than the comparison day. The interaction effect time*day was not significant (F = 1.79, *p* = 0.184), meaning that cortisol levels decreased in a similar fashion on both days (Fig. 3). Descriptive results for all measures of physiological and psychological stress can be found in Table 3.

**Table 3:** Measures of central tendency of all measures of physiological and psychological stress

Cortisol (nmol/l) M(SD)				Agitation M(SD)			Worry M(SD)			STAI-SKD M(SD)		
T0	T2	T0H	T2H	T0	T1	T2	T0	T1	T2	T0	T1	T2
4.09 (4.97)	3.73 (3.98)	2.66 (3.59)	2.09 (2.45)	28.56 (26.15)	24.17 (23.96)	16.73 (22.19)	28.93 (26.65)	25.22 (24.60)	17.32 (21.17)	1.81 (0.68)	1.77 (0.63)	1.51 (0.50)

*STAI-SKD* state anxiety, *T0* Pre-MRI, *T1* Post-Intervention, *T2* Post-MRI, *T0H* Home sample at time of T0, *T2H* Home sample at time of T2

### Primary analyses

#### Differences between the experimental groups depending on type of preparation (information vs. relaxation)

**Fig. 4:**
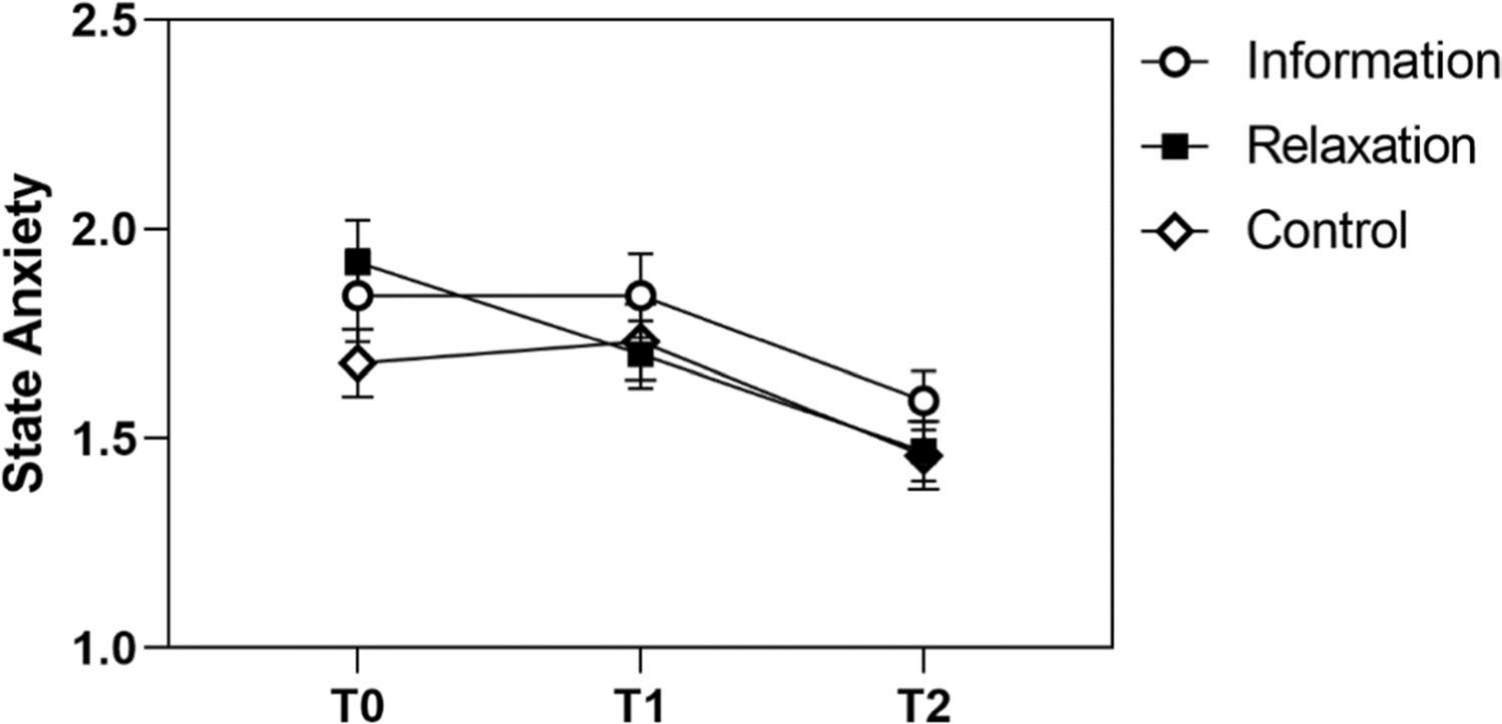
STAI-SKD levels of the information, relaxation, and control group

We then asked whether patient preparation through the information or relaxation video improved the patients’ psychological and physiological response. The mixed ANOVAs for all outcomes were characterized by a main effect of time, that is, declining levels from pre- to post MRI, except for cortisol. The results of this and the following analyses are displayed in Table 4. There was no main effect of group on any outcome and no interaction of group*time on agitation, worry, and cortisol. The *p*-value for the group*time interaction on state anxiety was *p* = 0.076 (Fig. 4); separate rmANOVAs for each experimental group revealed a significant main effect of time in all three groups (all F > 7.57, all *p* < 0.004). This was qualified by a linear and quadratic trend in the information and control group (linear: all F > 7.66, all *p* < 0.008; quadratic: all F > 6.33, all *p* < 0.015), but only a linear trend in the relaxation group (linear: F = 21.82, *p* < 0.001; quadratic: F < 0.01, *p* = 0.964). In the latter, state anxiety decreased in a linear fashion already before the MRI examination; in the information and control group, state anxiety rather showed a pronounced decline only after MRI.

**Table 4:** Results of the different ANOVAs

	Cortisol		State anxiety		Agitation		Worry	
	F	*P*	F	*p*	F	*p*	F	*P*
*Differences between experimental groups*								
Time	2.26	0.135	29.29	< 0.001	17.26	< 0.001	22.29	< 0.001
Group	< 0.01	0.998	0.84	0.433	0.35	0.703	0.32	0.724
Time*Group	0.60	0.550	2.35	0.076	0.62	0.598	1.12	0.341
Differences between experimental groups * coping styles								
Time	1.43	0.235	15.25	< .001	8.48	0.001	14.37	< 0.001
Group	0.45	0.637	1.25	0.290	1.19	0.309	0.90	0.411
Coping style	0.39	0.764	1.20	0.312	0.23	0.877	1.33	0.270
Time*Group	0.24	0.785	1.37	0.257	0.69	0.553	0.43	0.733
time*coping style	2.08	0.108	0.31	0.883	0.57	0.753	0.60	0.680
Group*Coping style	0.57	0.757	1.03	0.412	1.14	0.346	0.52	0.792
Time*Group*Coping style	0.99	0.437	1.36	0.215	0.99	0.451	0.82	0.597
*Differences between groups of congruence*								
*Overall analysis*								
Time	2.34	0.129	25.36	< .001	17.37	< 0.001	21.61	< 0.001
Congruence	1.69	0.189	2.67	0.074	0.40	0.673	1.36	0.262
Time*Congruence	2.49	0.088	3.00	0.035	2.42	0.071	2.37	0.071
*Congruent*								
Within (Time)	2.36	0.142	13.93	< 0.001	10.63	0.001	12.49	< 0.001
Linear contrast	–	–	17.50	< 0.001	13.04	0.002	16.42	< 0.001
Quadratic contrast	–	–	0.158	0.696	2.28	0.150	0.54	0.473
*Incongruent*								
Main effect	0.29	0.635	4.80	0.022	2.10	0.142	6.33	0.004
Linear contrast	–	–	6.22	0.016	–	–	8.59	0.005
Quadratic contrast	–	–	1.35	0.251	–	–	1.72	0.196
*Control*								
Main effect	0.023	0.880	10.69	< 0.001	9.60	0.001	4.98	0.022
Linear contrast	–	–	7.65	0.008	10.77	0.002	4.82	0.034
Quadratic contrast	–	–	17.70	< 0.001	6.53	0.015	5.43	0.025

#### Differences between the experimental groups depending on type of preparation (information vs. relaxation) and coping style

**Fig. 5:**
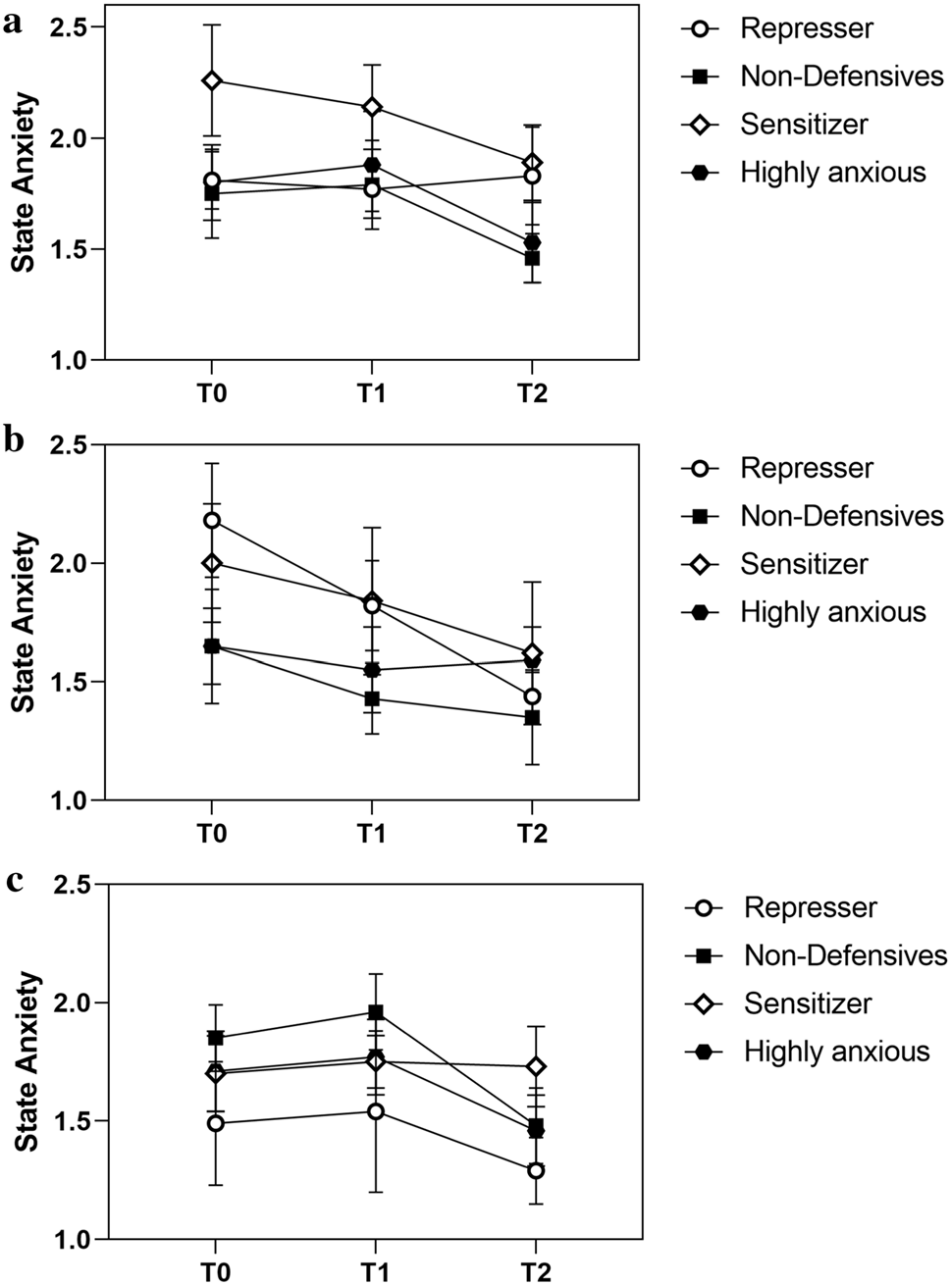
STAI-SKD levels of the different coping styles in the information (**a**), relaxation (**b**), and control group (**c**)

To analyze the effect of congruent patient preparation, we determined the patients’ coping style as described above using cluster analysis, then mixed ANOVAs were applied (within-subjects factor: time, between-subjects-factors: coping style, experimental group). Table 2 depicts the distribution of patients of the different coping modes to the three experimental groups. There was a significant main effect of time in the mixed ANOVAs for all outcomes except for cortisol but no significant main effect of group, coping style, or interaction effect on any outcome. That means that levels of the psychological markers decreased from pre- to post-MRI, but there was no significant difference between the different groups and / or coping styles regarding the general levels of the outcomes or their development over time. Figures 5a–c illustrate the development of the patients’ state over time in the three experimental groups and dependent on their coping style exemplarily for state anxiety.

We then combined patients who had been prepared congruently (repressers in relaxation group; sensitizers in information group) vs. incongruently (all other combinations) to increase statistical power. Separate mixed ANOVAs for agitation, worry, and state anxiety again revealed a significant main effect of time, that is, relief from pre- to post-MRI. There was no main effect of time on cortisol. Further, there was no main effect of congruence on agitation, worry, and cortisol and only a trend on state anxiety. Further, there was a significant interaction effect congruence*time for STAI-SKD; for agitation, cortisol, and worry the *p*-values were < 0.10.

**Fig. 6:**
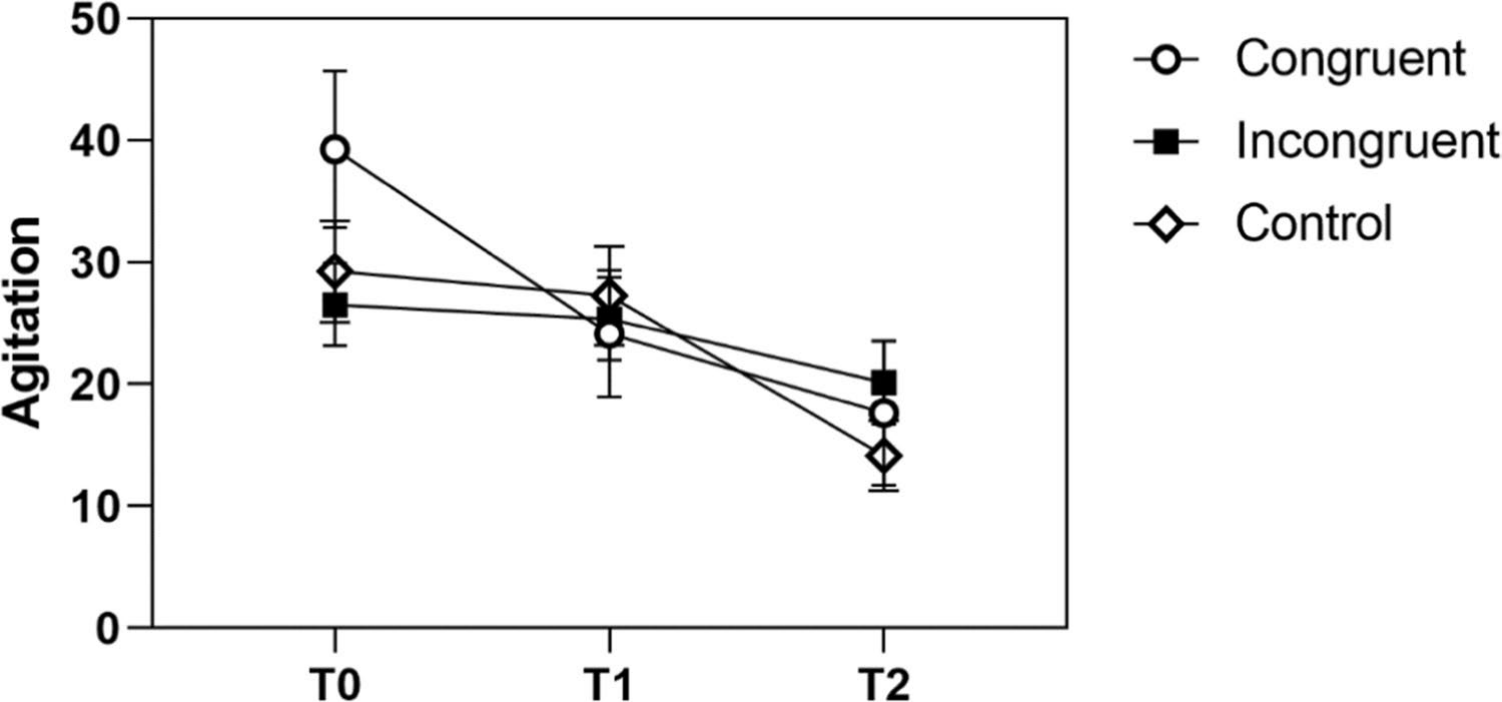
Agitation levels of the congruent, incongruent, and control group

Separate rmANOVAs for the congruent, incongruent, and control group revealed a significant main effect of time on agitation for the congruence and control group, which was not significant for the incongruent group. Specifically, the congruent group showed a linear but no quadratic contrast and the control group both. That means, that the congruent group experienced a pronounced decline of agitation after the intervention already which continued through the examination, while the decline was only slight during the waiting period in the control group but accelerated post-MRI. In the incongruent group, there was no significant decline at all (Fig. 6).

For worry, we found a significant main effect of time in all groups. This was qualified by a linear trend in all three groups (all F > 4.82, all *p* < 0.034); the quadratic trend was significant in the control group but not the congruent or incongruent group. Worry decreased over time in all three groups; however, this decrease was rather linear in the congruent and incongruent group, while patients in the control group only experienced relief of worry after the MRI scan.

**Fig. 7:**
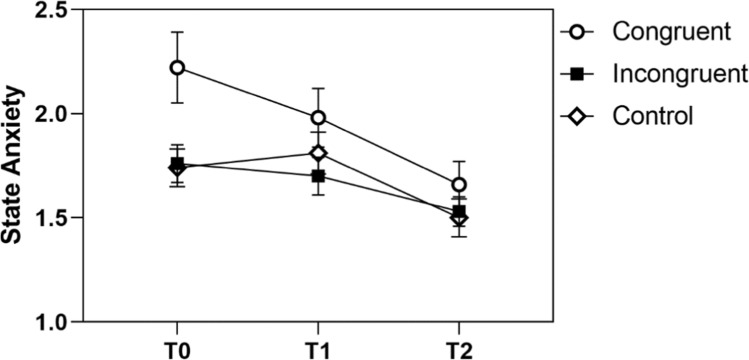
STAI-SKD levels of the congruent, incongruent, and control group

For state anxiety, there was a significant main effect of time in all three groups. The congruent group and the incongruent group showed a linear but no quadratic contrast, the control group both. Figure 7 shows that the control group experienced an increase from T0 to T1, but a decrease to T2; the congruent group experienced a pronounced linear decline of anxiety after the intervention already which continued through the examination; the decline was only slight during the waiting period in the incongruent group but accelerated slightly to post-MRI.

**Fig. 8:**
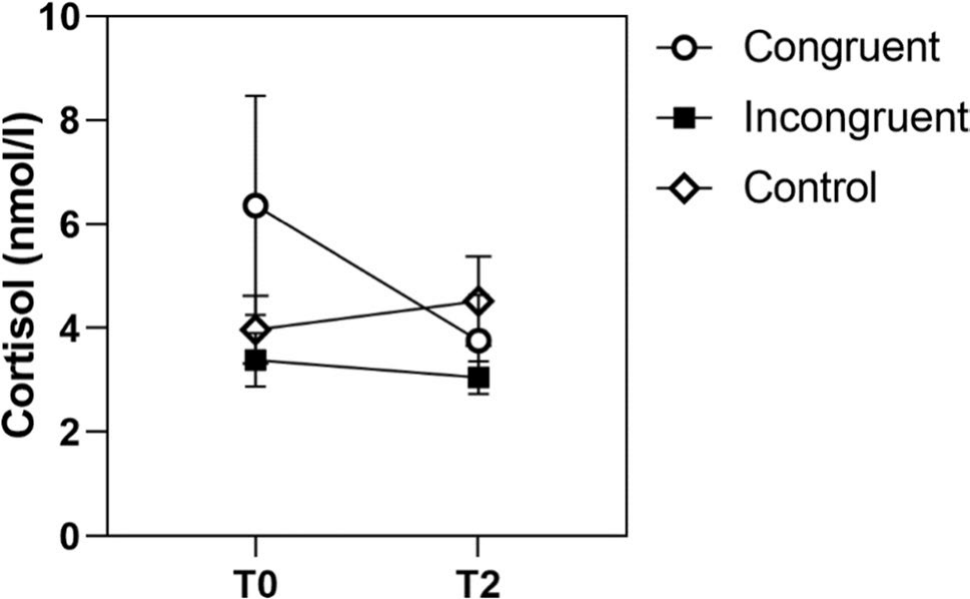
Cortisol levels of the congruent, incongruent, and control group

For cortisol, there was no main effect of time in any of the three groups, meaning that there was no significant change in cortisol in any of the groups (Fig. 8).

In sum, the pattern shows most consistently that patients with congruent preparation experienced relief on the psychological markers after the intervention already. In patients with incongruent preparation, these results are less consistent; in the case of agitation, there was no significant decline at all. Last, it can be summarized that patients of the control group only experienced a pronounced decline in anxiety after the MRI examination.

## Discussion

In this study we sought to analyze the effect of two video-based interventions aimed at preparing patients for MRI on the patients’ psychological and physiological response to the MRI examination. In general, patients’ cortisol levels were higher on the day of MRI compared to an individual no-stress baseline, which indicates that patients experienced distress in anticipation of their examination. Neither the informational nor the relaxational intervention had a consistent positive effect on the patients’ response. Yet, when patients were prepared according to their coping style they tended to relax after the intervention and before the MRI scan already while those patients with no additional preparation only experienced this relief after the scan and results were ambiguous for the incongruent preparation group. Research on the connection of patient anxiety or stress and clinical processes suggests that improving patient experience may also have a positive impact for healthcare operations.

This is the first study to compare cortisol levels of MRI patients to an individual no-stress baseline; we found cortisol levels to be elevated at the day of MRI which mirrors the mild to moderate levels of psychological tension that patients reported on average. The fact that we also found decreasing cortisol levels from pre- to post-MRI contradicts previous research that reported a rise or no significant change (Derntl et al., 2015; Madl et al., 2022; Tazegul et al., 2014). Taken together, this indicates that patients in fact do experience distress in the context of MRI. Thereby distress seems to be greatest in anticipation of the examination. The fact that cortisol-levels decreased from pre- to post-MRI indicates that the stress induced by the examination itself seems to be of minor importance. This blends in with results regarding the well-established decrease of psychological distress and anxiety from pre- to post-MRI (Dantendorfer et al., 1997; Katz et al., 1994; Mackenzie et al., 1995); it also extends findings from previous studies which had not been able to ascertain whether a lacking change of cortisol throughout the examination was due to a lack of distress or caused by anticipation and maintenance effects (Derntl et al., 2015; Madl et al., 2022).

One major goal in this study was to determine whether the informational and relaxation intervention improved the patients’ psychological and physiological reaction to MRI. The data only partly supported this hypothesis: The information, relaxation, and control group did not differ in the response pattern of cortisol, agitation, or worry. All groups experienced relief after the examination. However, there was a trend that patients of the relaxation but not of the information or control group tended to experience a relief of anxiety after the intervention, already. Providing a relaxational intervention therefore might be somewhat beneficial in terms of calming patients during the waiting period, which is in line with previous findings (Munn & Jordan, 2013). Relaxational interventions may be more beneficial in terms of reducing stress and anxiety in MRI patients, at least at the time point directly before the examination. The informational intervention did not have a positive effect on the patients’ reaction, which is in line with other studies reporting a lacking or even negative effect of merely informational interventions (Munn & Jordan, 2013; Quirk et al., 1989; Törnqvist et al., 2006). Ahlander et al. (2018) suggested that patients might be in a suboptimal state of susceptibility for information when already in direct anticipation of the examination. This is supported by studies in children that showed that processing information takes a relatively long time until it can serve as a basis for successful coping (Manne et al., 1990, 1992). Thus, providing information in advance (e.g., when scheduling the upcoming MRI), might lead to different results in terms of reducing pre-examinational stress and anxiety. Similarly, Phillips and Deary (1995) suggested that relaxation techniques also require practice; therefore, providing patients with preparational material of any kind in sufficient advance might be a reasonable approach. Apart from these general considerations, a more detailed view on the underlying reasons for MRI-related anxiety and the individual coping style might be decisive and will be discussed in the following section.

We had hypothesized that patient preparation congruent to individual coping style would lead to the most pronounced decrease of stress and anxiety. This hypothesis was not supported in the detailed analysis for all coping styles, probably due to issues of statistical power. However, combining those who had been prepared congruently or not congruently fully supported our hypothesis for state anxiety; for agitation and worry results were less clear. All patients were less anxious after the examination, but the congruent group experienced a relief of anxiety during the waiting period already. These findings are in line with studies conducted in other medical settings which also reported the most positive outcomes regarding anxiety, pain, and adaptation in patients who had been prepared according to their coping style (Krohne & El-Giamal, 2008; Ludwick-Rosenthal & Neufeld, 1993; Martelli et al., 1987; Morgan et al., 1998). This finding contributes to explaining why previous studies on informational and relaxational interventions have revealed inconsistent results (Munn & Jordan, 2013): Fit of coping style with type of patient preparation might be crucial for the respective interventions to produce positive effects. Patients fear different aspects of MRI that may mirror different coping styles: For some, the uncertain situation and lack of knowledge about it is paramount (vigilance), whereas others rather fear the MRI scanner itself or the results of the examination (avoidance; Carlsson & Carlsson, 2013; Dantendorfer et al., 1997; Katz et al., 1994; Thorpe et al., 2008). Lastly, feelings of helplessness and lack of control could reflect both: Uncertainty due to lack of information or arousal tracing back to the unalterable circumstances of undergoing MRI (e.g., constriction, not being able to move). Building upon these considerations and our results, patient preparation congruent with coping style might indeed have the potential to alleviate stress and anxiety more effectively than standardized or no patient preparation. When targeting patient preparation is not possible, incongruent preparation by relaxation seems to be less detrimental than by information: Although mostly being reported to reduce anxiety (Munn & Jordan, 2013), Törnqvist et al. (2006) even found negative effects for merely informational preparation. This is in line with findings from other medical contexts, where additional information on one’s disease induced detrimental effects for those with an avoidant coping style (Dahle Ommundsen et al., 2022; de Rooij et al., 2019). In contrast, results for relaxational interventions are generally positive and only vary in effect size (Munn & Jordan, 2013). When analysis of coping style is not possible, relaxational interventions therefore might be the safer way to go. Yet, the best option might be to offer a broad variety of preparation material for patients to choose from and best in advance. This has even two advantages: 1) patients can choose what fits their needs best and through this, 2) their sense of control is strengthened. This is particularly important as loss of control is one of the factors associated with MRI-related anxiety (Carlsson & Carlsson, 2013). Anxiety and physiological stress markers have been linked to unexpected patient behaviors like motion artifacts and premature terminations, thereby impacting operational efficiency (Dewey et al., 2007; Dziuda et al., 2019; Enders et al., 2011; Madl et al., 2022; Nguyen et al., 2020; Powell et al., 2015). Thus, we hypothesize that if we manage to address each patients’ needs optimally, there also might be a positive effect on operational smoothness for the medical institutions.

Despite our sample size of 142 patients, missing responses and the random distribution of patients to the experimental groups lead to a relatively low number of patients of each coping style per group. This limited the statistical power for our analyses of congruence; the hypothesized effects could only be observed on a general level of congruent preparation but not for sensitizers and repressers individually. Further, despite thorough randomization, the congruent patient group had slightly higher levels of state anxiety than the other groups. One might criticize that the steeper decline throughout the waiting period might trace back to statistical regression. Yet, one might as well argue that congruent preparation even managed to achieve a significant decrease although the patients were most anxious beforehand. Lastly, we were able to distinguish patients with the four coping styles through cluster analysis. Still, the levels of cognitive avoidance were higher than those of sensitization in all clusters. This finding of higher levels of cognitive avoidance in the medical context has been described previously (Sturmbauer et al., 2019). Sturmbauer et al. (2019) postulate that engaging in avoidant coping strategies might be particularly adaptive in the medical setting where the individual possibilites to exert control on the situation are very limited, rendering vigilant behavior rather unsuitable. Accordingly, the sensitizers in our study still exhibited relatively high levels of cognitive avoidance which might have limited the potential positive effect of the informational intervention. This again speaks in favor of the notion that it is beneficial to provide information in advance when patients still perceive a greater scope of action.

Despite these limitations, this study makes an important contribution for exploring the importance of interindividual differences in patient preparation. Our findings imply that uniform approaches to enhance patient education on an exclusively informational level fall short for adequate patient preparation and improvements of patient experience. Instead, healthcare providers should also consider ways to meet patients’ tendency to engage in cognitive avoidance, especially bearing in mind the higher levels of this strategy in the medical context. Further, it should be emphasized that educational material should best be applied in sufficient advance when patients still are more receptive and the same might be advisable for other interventions, as well. Assessing potential differences depending on the timing of intervention therefore may constitute an important area for future research. Last, efforts to improve patient experience should always consider interindividual differences that influence the patients’ way of coping with stressful situations in the medical context. There probably is no “one-size-fits-all” solution for optimizing patient experience; instead, individual differences should be taken into account.

## Data Availability

Data and material is available on reasonable request.
